# Impaired breathing, sleeping, vitality, and depression, and negative impact of L-T4 treatment characterize health-related quality of life in older people with stable CVD

**DOI:** 10.1007/s40520-020-01537-9

**Published:** 2020-04-10

**Authors:** Anna K. Ojala, Harri Sintonen, Risto P. Roine, Timo E. Strandberg, Camilla Schalin-Jäntti

**Affiliations:** 1 Endocrinology, Abdominal Center, Helsinki University Hospital and University of Helsinki, P.O. Box 340, 00290 Helsinki, Finland; 2 Department of Public Health, University of Helsinki, Helsinki, Finland; 3 Group Administration, University of Helsinki and Helsinki University Hospital, Helsinki, Finland; 4 Department of Health and Social Management, University of Eastern Finland, Kuopio, Finland; 5 University of Helsinki, and Helsinki University Hospital, Helsinki, Finland; 6 Center for Life Course Health Research, University of Oulu, Oulu, Finland

**Keywords:** HRQoL, CVD, Older people, TSH, L-T4

## Abstract

**Background:**

Cardiovascular disease (CVD) and thyroid dysfunction are common in older people, but little is known about how they affect health-related quality of life (HRQoL).

**Methods:**

We assessed HRQoL with the 15D instrument in 329 home-dwelling patients aged ≥ 75 years with stable CVD and compared the results to those of an age- and gender-matched general population (*n* = 103). We also studied the impact of age, BMI, number of medications, thyroid-stimulating hormone (TSH) concentration, levothyroxine (L-T4) substitution and Mini-Mental State Examination (MMSE) on HRQoL.

**Results:**

Overall HRQoL was impaired in older people with stable CVD (mean 15D score 0.777 vs 0.801, *p* = 0.001), and also on single dimensions of breathing, sleeping, discomfort and symptoms, distress, vitality (all *p* < 0.001), and depression (*p* = 0.016) compared to the age- and gender-matched general population. Furthermore, in the patients, L-T4 substitution associated with impaired sleeping (*p* = 0.018) and sexual activity (*p* = 0.030). Moreover, MMSE points, number of medications used, age (all *p* < 0.001) and BMI (*p* = 0.009) predicted impaired HRQoL.

**Conclusions:**

Older people with stable CVD are characterized by impaired HRQoL compared to age- and gender-matched controls. We demonstrate that this is the consequence of impaired breathing, sleeping, discomfort and symptoms, distress, vitality, and depression. L-T4 substitution has a negative impact on HRQoL in old patients with stable CVD. MMSE score, number of medications, age and BMI predict worse HRQoL.

## Introduction

Cardiovascular disease (CVD) and mild thyroid dysfunction are common in older people. The prevalence of CVD (including coronary heart disease, heart failure, stroke and hypertension) is about 80% in men and women aged 60–79 years, and further increases with aging to about 90% in both sexes in individuals aged 80 years or older [1]. Even when hypertension is excluded, the prevalence of CVD in men and women aged 60–79 increases from 18–25 to 34–43% in individuals over 80 years [1]. Mild thyroid dysfunction is found in over 10% of older people, and the prevalence of subclinical hypothyroidism, i.e., mildly elevated thyroid-stimulating hormone (TSH) concentrations is even higher, i.e., 21% in women and 16% in men aged 74 years or older [2, 3]. In a recent meta-analysis, in nonpregnant adults with subclinical hypothyroidism, levothyroxine (LT-4) substitution did not improve general quality of life or possible thyroid-related symptoms [4]. In cardiovascular trials and studies on CVD, older people are commonly underrepresented [5]. Moreover, longevity itself does not routinely guarantee a good quality of life. Data on how stable CVD or mild thyroid dysfunction affect health-related quality of life (HRQoL) in older individuals is scarce. Previous population-based studies on CVD and HRQoL have been cross-sectional surveys, the focus has not been on older people with CVD, and has not included data on laboratory and clinical examination of the study participants [6–9]. Furthermore, the factors accounting for possible impaired HRQoL have not been identified. We, therefore, wanted to study HRQoL in a cohort of older people (> 75 years) with stable CVD with the 15D instrument for assessment, not only of overall HRQoL, but also of 15 different single dimensions, and compare the results to those of an age- and gender-matched general population. Within our cohort of old people with stable CVD, we further assessed the possible relationships of TSH concentrations and L-T4 substitution with HRQoL. We also searched for possible predictors of impaired HRQoL within the patient group.

## Design and methods

### Study population and controls

These are secondary analyses of the prospective Drugs and Evidence Based Medicine in the Elderly (DEBATE) study [10], a geriatrician-internist managed, real-life trial originally aimed at investigating the effect of multifactorial cardiovascular prevention in patients aged 75 years or older with stable CVD. Stable CVD was defined as having prior myocardial infarction, coronary artery disease, peripheral artery disease, previous stroke or transient ischemic attack, from which the patient had recovered. This population-based study consisted of 400 home-dwelling patients aged 75 years or older living in Helsinki, Finland, who were randomly recruited using pre-study mailed questionnaires. All study participants had confirmed CVD, mostly accounted for by coronary heart disease (82%).

Controls for HRQoL comparisons were obtained from the Finnish National Health 2000 Health Examination Survey [11]. In the original study, a two-stage stratified cluster sampling was performed to obtain a representative cohort of the whole population aged 30 years or older. The age group of 80 or over was oversampled to ensure a sufficient number of older participants, which was also taken into account in the data analysis. Initially, the survey was carried out in different phases, including comprehensive health interviews and examinations, telephone interviews and questionnaires. For the current study, a control population in the age range of the patients and living in the Helsinki University Hospital catchment area was selected and weighted to correspond to the age and gender distribution of the patients.

### Measurements

In older people with stable CVD, clinical status and laboratory parameters (performed with routine methods in the central laboratory of the Helsinki University Hospital) were assessed yearly beginning from year 2000. TSH measurements (*n* = 327; reference range for TSH 0.5–3.6 mU/l [12]) were performed in 2002. Cognitive performance was evaluated using the Mini-Mental State Examination (MMSE) tool, and The Zung Self-Rating Depression Scale questionnaire for screening of depression. In years 2000 and 2002, HRQoL was assessed with the 15D instrument in 329 of the study subjects (F/M = 216/113 aged 82.8 ± 4.7 years) and further compared to an age- and gender-matched sample of the general population (*n* = 103). We further compared the HRQoL of the DEBATE study population to the general population adjusting for diabetes, hypertension, myocardial infarction, heart failure, angina, stroke and claudication. Moreover, in the DEBATE cohort, comparisons, adjusted for age, gender, MMSE points and education (primary school or higher), between the 15D scores were made between different TSH ranges (TSH < 0.5 mU/l; TSH = 0.5–3.6 mU/l; TSH > 3.6 mU/l), as well as for those on L-T4 substitution compared to those not receiving L-T4 substitution. Finally, in the patient group, the 15D scores recorded in 2000 were compared to the 15D scores measured in 2002. We also searched for possible predictors of HRQoL in patients with stable CVD, such as age, BMI, marital status, level of education, number of medications and MMSE scores.

### Mini-mental state examination

The Mini-Mental State Examination (MMSE), originally developed for assessing cognitive performance in psychiatric patients [13], is commonly used in cognitive function testing among older individuals. The test consists of evaluation of attention, orientation, memory, calculation, recall, registration and visuo-spatial skills scored from 1 to 30. The cut-off point most commonly used is 24, lower scores reflecting worse performance. The MMSE is used in clinical practice as a screening test for dementia and also in assessment of suspected dementia. However, although the MMSE contributes to diagnosis of dementia, it is recommended not to be used as a single exclusion or confirmation test of dementia [14].

### The Zung self-rating depression scale

The Zung self-rating depression scale is a 20-item, self-administered, Likert scale used for screening of depressive symptoms [15]. Each item includes a 4-point scale, ranging from none or a little of the time (1) to most or all of the time (4). For evaluating the severity of depressive symptoms, raw scores (range 20–80) are converted into index scores that range from 25 to 100. The most conventionally used cut-off point is index score 50, higher index scores indicate more clinically relevant symptoms.

### The 15D instrument in assessing health-related quality of life

The 15D instrument is a generic, well-validated, self-administered tool for measuring HRQoL in individuals aged 16 or older [16]. It consists of 15 dimensions: mobility (move), vision (see), hearing (hear), breathing (breath), sleeping (sleep), eating (eat), speech, excretion (excret), usual activities (uact), mental function (mental), discomfort and symptoms (disco), depression (depr), distress (distr), vitality (vital) and sexual activity (sex). It can be used both as a profile and a single index score measure of HRQoL. Each dimension includes five levels from which the respondent chooses the most appropriate one to describe his/her present health status. For HRQoL assessment, a 15D score is generated over all the dimensions on a 0–1 scale, higher scores reflecting better HRQoL. The 15D provides a highly reliable, sensitive and generalizable tool for assessing HRQoL and it is also valid for deriving quality-adjusted life years (QALYs) [17–19]. The minimum clinically important cross-sectional difference in the 15D score is 0.015 [20].

### Statistical analyses

Statistical analyses were performed using SPSS statistical software v. 25.0 (IBM Corp.) by performing independent samples *t* test and linear regression analyses. For analysis of the correlations, the Pearson correlation coefficient was used. For detection of multicollinearity, tolerance statistics and variance inflation factor (VIF) were investigated. A *p* value < 0.05 was considered statistically significant.

## Results

### Characteristics of the study subjects

Clinical and laboratory data of the study subjects according to given TSH ranges (TSH < 0.5 mU/l; TSH = 0.5–3.6 mU/l; TSH > 3.6 mU/l) are presented in Table 1. There were no significant differences in age, sex, education (primary school or higher), MMSE points or Zung scale distribution between different TSH ranges. Thirty-six subjects (F/M = 36/0) received L-T4 replacement therapy. The number of users was highest within the normal reference range of TSH (TSH = 0.5–3.6 mU/l). Thirty-five percent of subjects with a suppressed TSH (< 0.5 mU/l) were on L-T4. Medication for cognitive disorder was used by four subjects.

**Table 1:** Clinical and laboratory data of older people with stable CVD (*n* = 327) according to different TSH ranges

Variables	TSH range < 0.5 mU/l (*n* = 20)	TSH range 0.5–3.6 mU/l (*n* = 261)	TSH range > 3.6 mU/l (*n* = 46)	*p* value between different TSH ranges	Total population (*n* = 327)
Age, mean (SD)	82.4 (4.9)	82.6 (4.8)	83.8 (4.7)	0.25	
Women, *n* (%)	15 (75)	168 (64.4)	32 (69.6)	0.53	215 (65.7)
Primary school only, *n* (%)	7 (36.8)	91 (35.0)	24 (52.2)	0.085	122 (37.3)
BMI				0.27	301
< 25, *n* (%)	4 (21.1)	94 (39.5)	14 (31.8)		112 (37.2)
25–29, *n* (%)	12 (63.2)	92 (38.7)	19 (43.2)		123 (40.9)
> 29, *n* (%)	3 (15.8)	52 (21.8)	11 (25.0)		66 (21.9)
TSH, mean (SD)	0.28 (1.16)	1.56 (1.13)	5.81 (1.09)	< 0.0001	
Thyroxin treatment, *n* (%)	7 (35.0)	23 (8.8)	6 (13.0)	0.0013	36 (11.0)
MMSE, mean (SD)	26.1 (14.3)	26.6 (14.5)	25.4 (14.2)	0.85	
Zung, mean (SD)	36.3 (8.9)	37.7 (9.7)	38.4 (8.8)	0.68	

### HRQoL in old patients with stable CVD compared to the general population

**Fig. 1:**
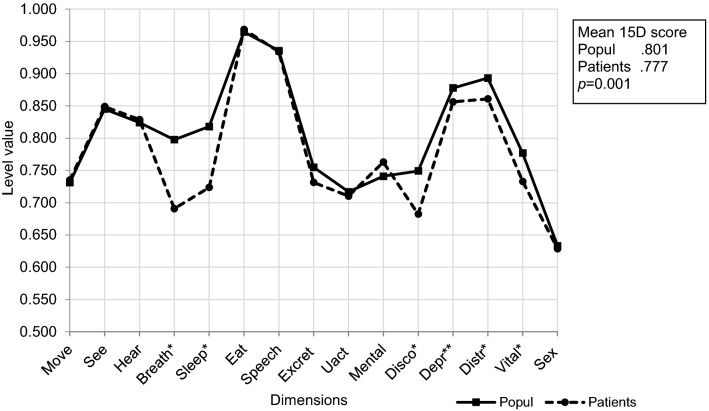
The mean 15D scores and profiles of old patients with stable CVD (*n* = 329) compared to the age- and gender-matched general population (*n* = 103). Statistically significant differences in 15D dimensions between the groups and corresponding *p* values are indicated with asterisks. *p < 0.001, **p = 0.016

Overall HRQoL was impaired in old patients with stable CVD compared to the age- and gender-matched general population (mean 15D score 0.777 vs 0.801, *p* = 0.001; Fig. 1). The observed difference between the mean 15D scores was also clinically important [20]. Significant impairments were observed on the dimensions of breathing, sleeping, discomfort and symptoms, distress, vitality (all *p* < 0.001), and depression (*p* = 0.016) (Fig. 1). In further analyses, this difference in overall HRQoL disappeared when the findings of the old patients with CVD were adjusted for diabetes, hypertension, myocardial infarction, heart failure, angina, stroke and claudication (*p* = 0.195, data not shown).

### Effect of 2-year aging on HRQoL and predictors of HRQoL in older patients with stable CVD

Mean 15D score recorded in 2002 did not differ statistically significantly from that obtained in 2000 (data not shown). In linear regression analysis (Table 2), variables predicting the 15D were MMSE points, number of medications used, age (for all *p* < 0.001) and BMI (*p* = 0.009) (Table 2). The adjusted *R*^2^ was 0.213. Sex, marital status, education, TSH level or L-T4 substitution did not influence overall HRQoL (data not shown). Signs of multicollinearity were not detected (data not shown).

**Table 2:** Linear regression analysis of variables predicting the 15D score in older patients with stable CVD

Predictor	Regression coefficient	*p*
Constant	1.149	< 0.001
MMSE points	0.009	< 0.001
Number of medications^a^	− 0.008	< 0.001
Age	− 0.005	< 0.001
BMI	− 0.004	0.009

^a^Levothyroxine treatment was not included in the total number of medications, since it was analyzed separately

### Relationship between HRQoL and thyroid function in older patients with stable CVD

**Fig. 2:**
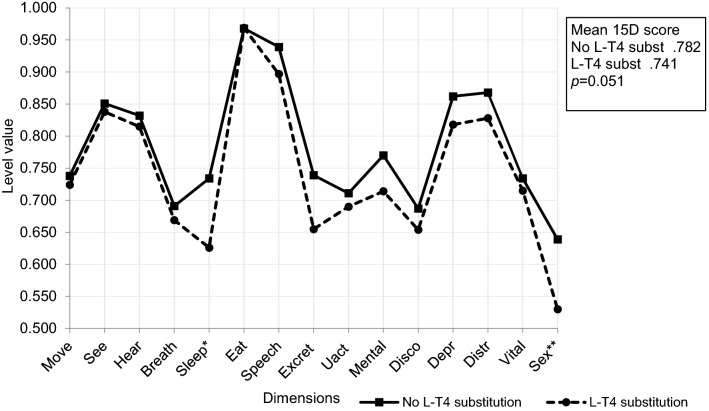
The mean 15D scores and profiles of patients on L-T4 substitution (*n* = 36) compared to that of subjects having no thyroid medication (*n* = 323). Statistically significant differences in 15D dimensions between the groups and corresponding *p* values are indicated with asterisks. *p = 0.018 **p = 0.030

No significant differences were found when the mean 15D scores or the single dimensions of HRQoL were compared between different TSH ranges. Subjects on L-T4 scored significantly worse on the dimensions of sleeping (*p* = 0.018) and sexual activity (*p* = 0.030) (Fig. 2).

## Discussion

We here demonstrate that old people with stable CVD are characterized by impaired HRQoL compared to the general population, and that this is due to impairments in the dimensions of breathing, sleeping, discomfort and symptoms, distress, vitality, and depression. Within the patient group, MMSE points, number of medications, age and BMI turned out as predictors of poorer HRQoL. During a 2-year follow-up, HRQoL remained stable in our cohort, indicating that HRQoL does not deteriorate rapidly in home-dwelling persons > 75 years. Furthermore, we also found that overall HRQoL is impaired in older people with stable CVD on L-T4 compared to those not on LT-4 therapy, although this was not statistically significant (mean 15D score 0.741 vs 0.782, *p* = 0.051). However, the difference of 0.041 is clinically important, as the minimum clinically important cross-sectional difference for the 15D score is 0.015 [20]. In addition, the dimensions of sleeping and sexual activity were both significantly impaired in subjects on compared to subjects without L-T4 therapy. Thyroxine substitution is thus not associated with improved well-being in older people with stable CVD. Moreover, in the present study, 35% of old patients with stable CVD characterized by suppressed TSH (< 0.5 mU/l) were on L-T4 therapy, indicating too large a dosing of L-T4.

Multimorbidity is common in older people, ranging from 55 to 98%. Its major consequences are risk of disability, functional decline and poor quality of life [21]. Moreover, the prevalence of both CVD and mild thyroid dysfunction increase with age [1, 2]. Our findings are in line with a previous population-based study, where HRQoL was impaired in patients with heart disease (angina, congestive heart failure or heart attack) on the domains of mobility and activities and participation in society compared to those with no prior heart disease, although congestive heart failure was generally associated with the greatest impairments in HRQoL [8]. However, our findings emphasize that in older individuals, stable CVD not only impairs overall HRQoL but also has a negative impact on multiple single dimensions of HRQoL compared to the general population. Of note, in a recent, prospective cohort study of 22,229 adults with no prior CVD, poor HRQoL associated with higher risk of incident CVD events (first non-fatal myocardial infarction (MI), fatal MI or coronary heart disease death, and fatal and non-fatal stroke) [22].

Older people are at elevated risk for adverse effects of medication and polypharmacy. A large retrospective cohort study of 52,298 individuals demonstrated falling borderline elevated TSH thresholds for initiating LT-4 therapy as median threshold TSH decreased significantly from 8.7 to 7.9 mU/l between years 2001 and 2009 [23]. Somewhat surprisingly, the odds ratio for initiating L-T4 when TSH was ≤ 10 mU/l was highest for older individuals and individuals with cardiac risk factors [23]. This led to suppressed TSH concentrations in 5.8% of individuals 5 years later, with TSH < 0.1 mU/L.

Our study revealed that a significant proportion of older people with stable CVD and suppressed TSH (< 0.5 mU/l) received LT-4 substitution. Likewise, in a previous study of patients aged 65 years or older on LT-4 substitution, up to 41% were characterized by decreased TSH concentrations [24]. According to the 2013 guideline of the European Thyroid Association on management of subclinical hypothyroidism the L-T4 starting dose should be small (25 or 50 µg daily) and treatment target for serum TSH higher (up to 5 mU/l) in patients with cardiac disease/patients > 70 years compared to younger subjects not suffering from cardiac disease [25].

In a recent randomized, double-blind, placebo-controlled study of subjects aged 65 years or older with subclinical hypothyroidism, L-T4 substitution was not associated with beneficial effects [26]. There is growing evidence that minor TSH elevations are not associated with impaired quality of life, symptoms, cardiovascular events or mortality in older population [27]. Our results are consistent with current recommendations against levothyroxine therapy in asymptomatic older persons with TSH concentrations < 10 mU/l [28].

Strengths of the study are that it is based on a geriatrician-internist managed, real-life, community-living cohort and that it includes both laboratory and clinical examination of the participants. The rather small control population (*n* = 103) can be regarded as a limitation of the study. However, to increase the specificity of our findings, we wanted to match the controls, not only regarding age- and sex, but also regarding the catchment area. This decreased the number of controls. However, as significant differences between the patient and the control population were found both in the total 15D score as well as in several single dimension scores, the findings would probably have been even stronger had the control population been larger.

## Conclusions

Compared to the age- and gender-matched general population, overall HRQoL and the dimensions of breathing, sleeping, discomfort and symptoms, distress, vitality, and depression are impaired in 75+ people with stable CVD. Levothyroxine substitution has a negative impact on HRQoL in older patients with stable CVD.
